# Nonenhanced CT-Based radiomics model enhances PTC detection in Hashimoto’s thyroiditis

**DOI:** 10.1186/s12885-025-15206-5

**Published:** 2025-11-12

**Authors:** Yun Peng, Kaiyao Huang, Zijian Gong, Wenying Liu, Jidong Peng, Lianggeng Gong

**Affiliations:** 1 Department of Radiology, The Second Affiliated Hospital, Jiangxi Medical College, Nanchang University,, Nanchang, 330006 China; 2 Intelligent Medical Imaging of Jiangxi Key Laboratory, Nanchang, 330006 China; 3 Department of Medical Imaging, Ganzhou People’s Hospital, The Affiliated Ganzhou Hospital of Nanchang University, Ganzhou, 341000 China

**Keywords:** Hashimoto’s thyroiditis, Papillary thyroid carcinoma, Radiomics, Nonenhanced CT, Machine learning

## Abstract

**Background:**

Hashimoto's thyroiditis (HT) is a common benign thyroid disease that often coexists with papillary thyroid carcinoma (PTC). Owing to the diffuse changes in the thyroid caused by HT, PTCs can be challenging to detect using conventional imaging modalities such as ultrasound and CT. The aim of this study was to develop a radiomics model based on nonenhanced CT (NECT) to predict the presence of PTC in the patients with HT, thereby improving early diagnostic accuracy.

**Materials and methods:**

This retrospective study included pathologically confirmed HT patients with or without PTC who underwent NECT scans within 30 days before surgery from January 2017 to April 2023 at Hospital I and Hospital II. The patients from hospital I were divided randomly at a ratio of 8:2 into a training cohort and an internal validation cohort. The patients from hospital II were assigned to the external validation cohort. Radiomic features were extracted using PyRadiomics. Intraclass correlation coefficient, Pearson correlation and LASSO analyses were conducted to reduce the dimensionality of the radiomic features. Four machine learning algorithms, including logistic regression (LR), naive bayes (NB), support vector machine (SVM), and multilayer perceptron (MLP) classifiers, were employed to develop and validate the prediction models based on the remaining features.

**Results:**

A total of 130 patients, 89 from Hospital I [71 in the training cohort and 18 internal validation cohort] and 41 from Hospital II [external validation cohort], were included. Six features with nonzero coefficients were retained by the LASSO algorithm for inclusion in the machine learning models. In the external validation cohort, the LR, NB, SVM, and MLP models obtained AUCs of 0.736, 0.690, 0.751 and 0.783, respectively. The MLP model performed the best in the external validation cohort, with an area under the curve of 0.783, a sensitivity of 0.643, and a specificity of 0.923.

**Conclusion:**

A radiomics model based on NECT could identify PTCs in patients with HT and had the potential to enhance early diagnosis and intervention for these patients.

## Introduction

Hashimoto’s thyroiditis (HT) is one of the most common benign thyroid disorders and is characterized by chronic lymphocytic infiltration and gradual destruction of thyroid tissue, often leading to hypothyroidism [1]. Studies have shown that HT is associated with and sometimes coexists with papillary thyroid carcinoma (PTC). Indeed, the incidence of PTC is greater in HT patients than in the general population [2] and 19% of PTC patients have been diagnosed with HT [3, 4]. The underlying mechanisms of this association are multifactorial and involve chronic inflammation, autoimmune processes, and potential genetic predispositions [5–7]. Guidelines dictate that HT patients usually need only regular follow-up or medication, and surgery is not required unless there is a concomitant malignancy or symptoms of compression. Identifying occult PTCs within the inflammatory milieu of HTs is crucial for decision making to support timely and effective management.

Currently, the gold standard for the diagnosis of PTC is still histopathological examination based on invasive fine-needle aspiration biopsy; however, biopsy sampling errors can lead to missed diagnosis of PTC [8]. The imaging methods, such as ultrasound and computed tomography (CT), although widely used, have limitations of difficult diagnosis only by eyes. HT typically presents as bilateral diffuse enlargement of the thyroid with heterogeneously decreased echo density [9], while PTCs commonly appear as nodules. When PTCs, especially micro-PTCs, coexist with HT, they are easily masked by diffuse thyroid changes caused by HT. Therefore, more advanced techniques and methods are needed to reduce missed detection of PTC.

Radiomics refers to the high-throughput extraction of quantitative features from medical images and subsequent construction of machine learning (ML) models with extracted features to predict genomic patterns and clinical outcomes. It has been widely applied in medical imaging analysis, especially in oncology [10, 11]. For example, Fang et al. suggested that ultrasound radiomics can distinguish between benign and malignant thyroid nodules in HT, confirming its role in analyzing the heterogeneity of thyroid lesions [12]. The purpose of this study was to develop a radiomics model to predict the presence of hidden PTC against the background of HT from nonenhanced CT (NECT).

## Materials and methods

### Patients

This retrospective study was approved by the institutional research ethics board, and the requirement for informed consent was waived owing to the retrospective nature of the study. Patients with pathologically confirmed HT who were treated at the Second Affiliated Hospital of Nanchang University (hospital I) and Ganzhou People’s Hospital (hospital II) from January 2017 to April 2023 were included, provided that they had undergone neck NECT examinations within 30 days prior to surgery to ensure imaging-surgical correlation and mitigate progression-related discrepancies [13]. The exclusion criteria included (1) poor-quality CT images or indistinct thyroid gland boundaries, and (2) a history of other malignancies.

### CT scanning

At hospital I, all neck NECT scans were obtained using a 128-slice machine (SOMATOM Definition Flash). For hospital II, all neck NECT scans were obtained using a 512-slice machine (Revolution CT; GE Healthcare). The parameters were as follows: 120 kV; automatic tube current; slice spacing, 5 mm; slice thickness, 5 mm.

### Segmentation of the region of interest and extraction of radiomic features

**Fig. 1:**
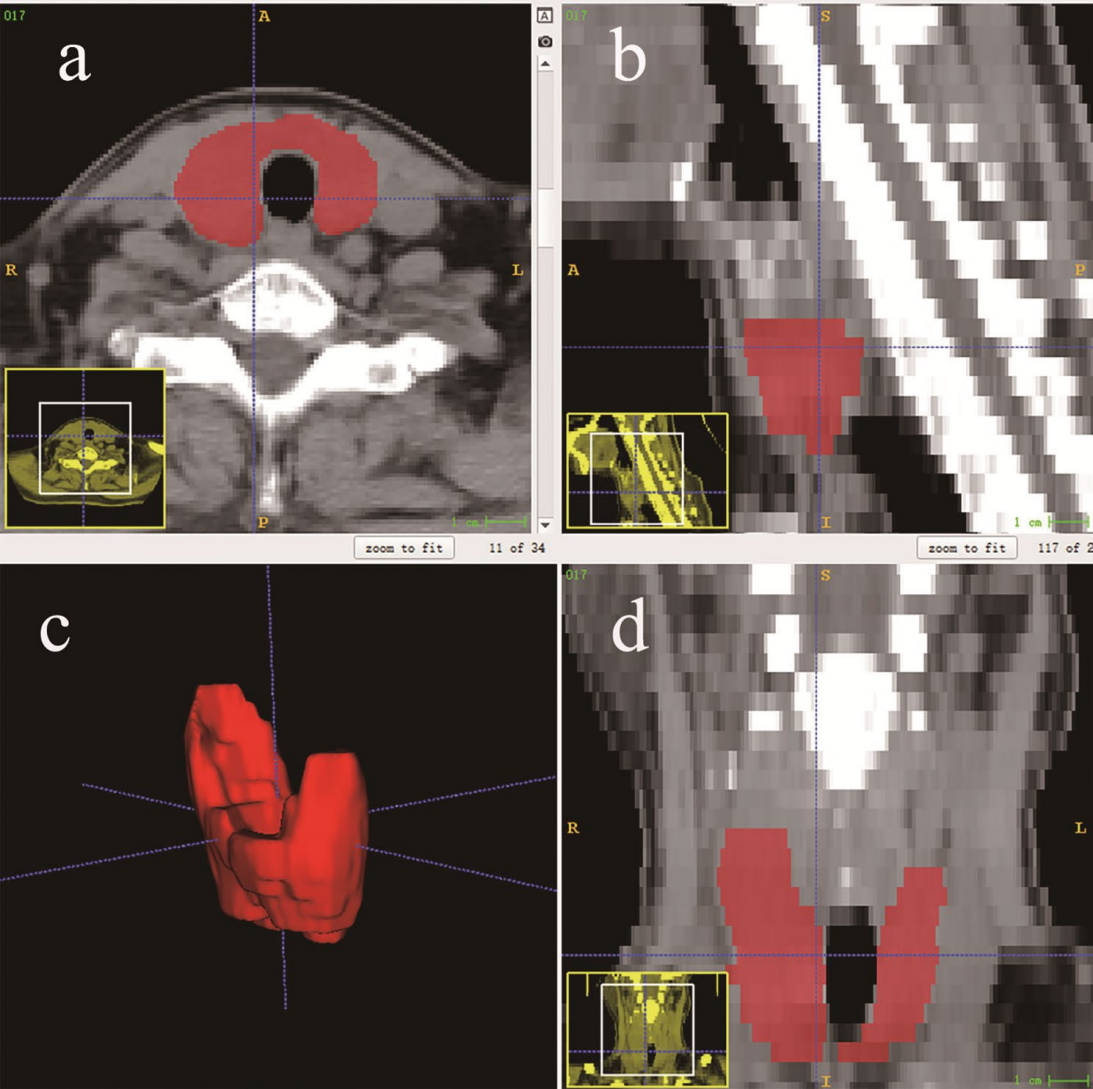
Schematic diagram of region of interest (ROI) placement for Hashimoto's thyroiditis. The ROI was manually drawn in red on the (**a**) axial, (**b**) sagittal, and (**d**) coronal CT images, generating a (**c**) 3-dimensional ROI.

In this study, preoperative NECT data were exported as DICOM files. Then, they were resampled to a 1 mm × 1 mm × 5 mm voxel size using 3D slicer software (version 5.2.2, https://www.slicer.org). The resampled CT images were then imported into ITK-SNAP software (version 3.8, http://www.itksnap.org/) to delineate regions of interest (ROIs). The delineation of each ROI was performed independently by two radiologists with 1 and 4 years of work experience. The ROI was drawn by outlining the edge of the thyroid and completely covering the whole gland (Fig. 1). Finally, radiomic data were extracted using the Python package PyRadiomics (version 3.0.1); a total of 851 features were extracted for each patient.

### Feature selection and model establishment

All the features were normalized to 0 ~ 1. To reduce the dimensionality of the radiomic features, we adopted the following procedure: First, the intraclass correlation coefficient (ICC) was used to estimate the reproducibility of each feature, and features with ICC values ≥ 0.75 were selected for further analysis. Second, a pairwise Pearson correlation analysis was used to identify pairs of features with an absolute correlation coefficient greater than 0.9; one feature from these pairs was selected at random for inclusion in further calculations. Third, the features were screened using least absolute shrinkage and selection operator (LASSO) analysis, and the optimal log (λ) value was determined through 10-fold cross validation.

Eventually, 4 ML models, including logistic regression (LR), naive Bayes (NB), support vector machine (SVM), and multilayer perceptron (MLP) classifier, were constructed based on the remaining features.

### Statistical analysis and model performance evaluation

Continuous features are reported as the means ± SDs, and categorical features are reported as counts and percentages. The age and sex of the three cohorts were compared using one-way analysis of variance (ANOVA) and the chi-square test, respectively. The models were evaluated via receiver operating characteristic (ROC) curve analysis to determine the area under the curve (AUC). Decision curve analysis (DCA) was conducted to determine the clinical usefulness of each model. SHAP analysis was performed to interpret the contributions of individual radiomic features in the best-performing machine learning model. SHAP summary dot plots were generated using the internal validation cohort to visualize feature importance. All statistical analyses were performed using IBM SPSS Statistics (Version 26), Python 3.7.12 (with scikit-learn 1.2.2, numpy 1.21.6, pandas 1.3.5, matplotlib 3.2.2, and SHAP 0.42.1), and R software (Version 4.1.3; www.r-project.org) supplemented with the packages rmda and rms. Statistical significance was defined as *p* < 0.05.

## Results

### Clinical characteristics

**Fig. 2:**
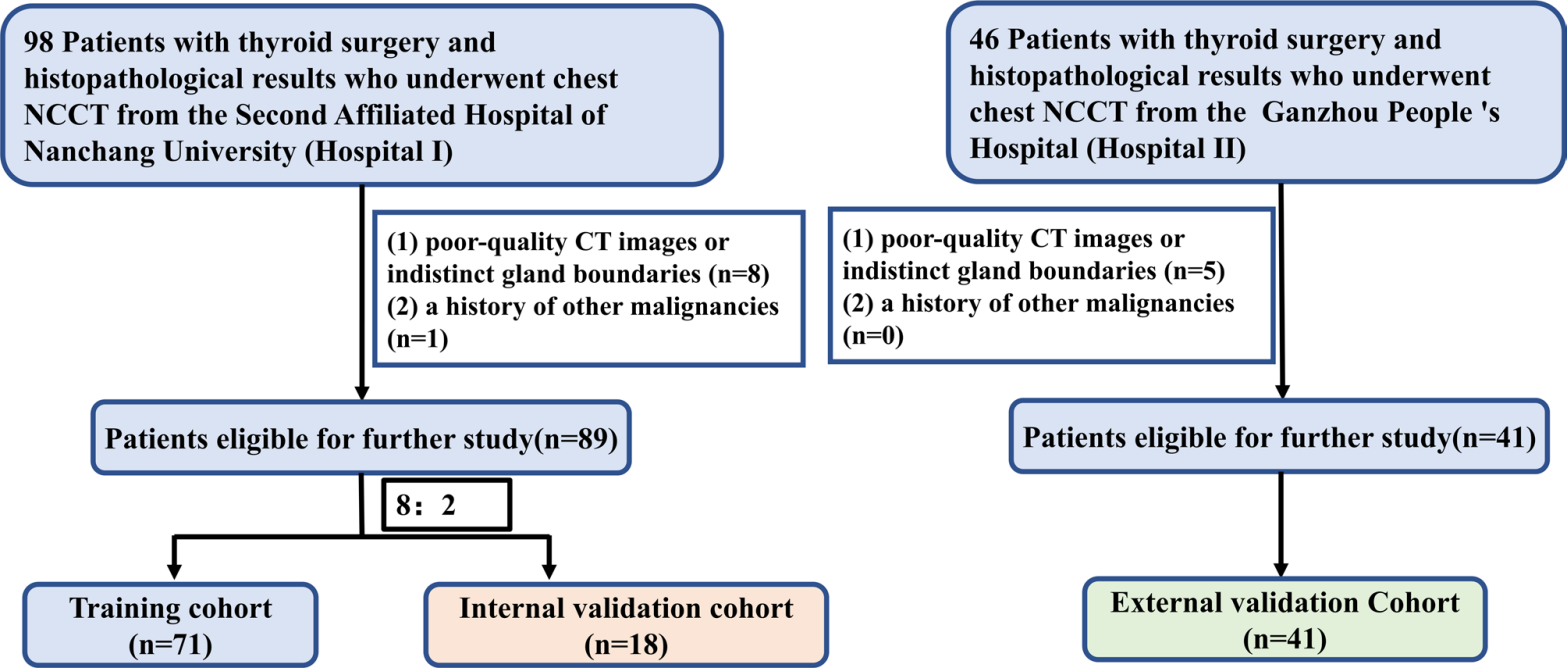
Recruitment of patients in our study

In accordance with the inclusion criteria, 98 patients from hospital I and 46 patients from hospital II were found. Among the patients from hospital I, 1 patient was excluded because of a history of other malignancies, and 8 patients were excluded because of poor-quality CT images or indistinct thyroid gland boundaries. Five patients from hospital II were excluded because of a lack of high-quality images. A total of 130 patients were ultimately included in the current study, with 89 from hospital I and 41 from hospital II (Fig. 2). The patients from hospital I were divided randomly at a ratio of 8:2 into a training cohort (*n* = 71) and an internal validation cohort (*n* = 18). The patients from hospital II were assigned to the external validation cohort (*n* = 41). The clinical characteristics of the three cohorts are summarized in Table 1. There were no significant differences among the three cohorts in any of the characteristics (all *P* > 0.05).

**Table 1:** Baseline data of the 3 cohorts

Characteristics	Training cohort (*n* = 71)	Internal validation cohort (*n* = 18)	External validation cohort (*n* = 41)	*P* value
Age (mean ± SD)	46.70 ± 14.75	42.89 ± 13.87	43.71 ± 10.27	0.38
Sex, n (%)				0.78
Male	3(4.2%)	1(5.6%)	3(7.3%)	
Female	68(95.8%)	17(94.4%)	38(92.7%)	
Histopathological result, n (%)				0.79
Without PTC	27(38.0%)	6(33.3%)	13(31.7%)	
With PTC	44(62.0%)	12(66.7%)	28(68.3%)	

### Feature selection

**Fig. 3:**
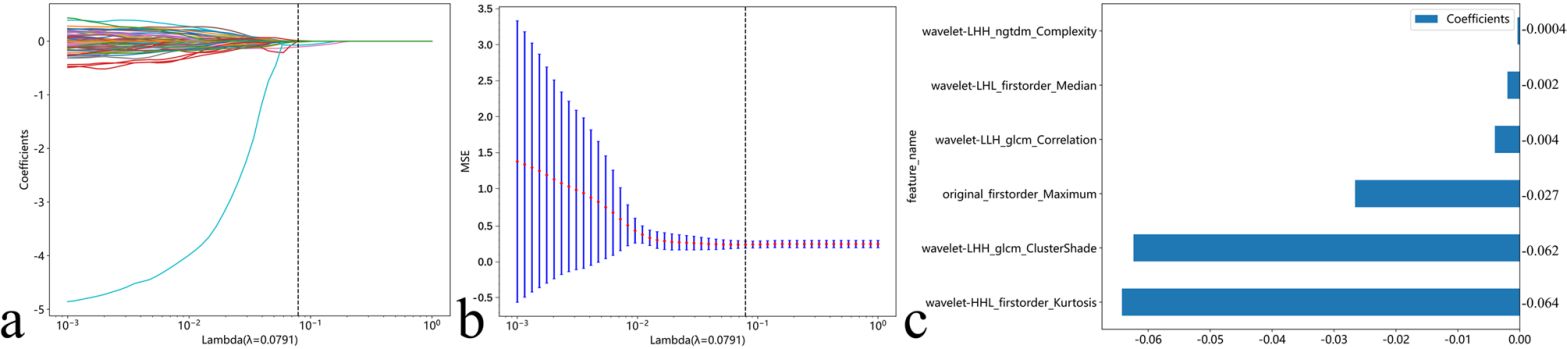
Feature selection using the least absolute shrinkage and selection operator (LASSO) algorithm. **a** Ten-fold cross-validation curve showing mean squared error versus log (Lambda), where the optimal Lambda (vertical dotted line) was determined by minimum criteria. **b** Coefficient trajectories across Lambda values, demonstrating how nonsignificant features were progressively eliminated (shrinkage to zero). **c** Final retained features with their nonzero coefficients at the optimal Lambda, presented as a labeled histogram for clarity

A total of 851 radiomic features were extracted for each ROI. Among them, 750 features (88.1%) with high reproducibility were retained. After Pearson correlation analysis, 163 features were selected. Finally, 6 features with nonzero coefficients were retained by the LASSO algorithm for further ML model construction. The details of the features are shown in Fig. 3.

### Model performance

**Fig. 4:**
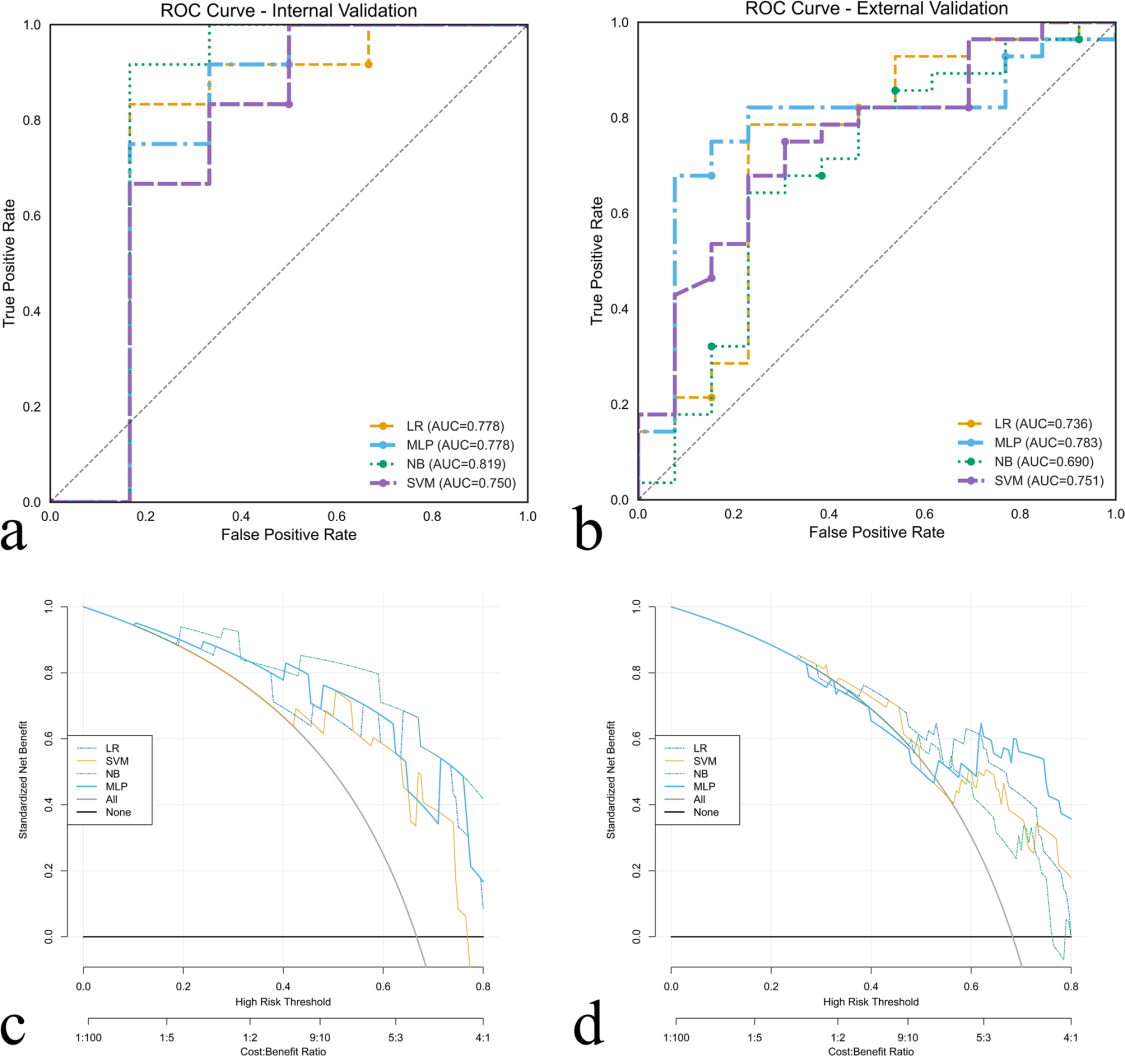
Receiver operating characteristic curves and decision curve analyses of the models in both the (**a**, **c**) internal validation cohort and (**b**, **d**) external validation cohort

Table 2 and Fig. 4 show the performance of the ML models. In the external validation cohort, the LR, NB, SVM, and MLP models had AUCs of 0.736, 0.690, 0.751, and 0.783, respectively. Among them, the MLP showed the best performance, with an AUC of 0.783 and a sensitivity and specificity of 0.643 and 0.923 (Fig. 4a and b), respectively, in the external validation cohort. The hyperparameters of MLP model was as follows: hidden_layer_sizes=(64, 64, 128, 128), max_iter=100, solver=’sgd’, random_state=0. The DCA of the models in the external validation cohort demonstrated that the models had demonstrated clinical net benefit over a range of threshold probabilities. The DCA of the MLP in the internal and external validation cohort revealed that the model was clinically useful (Fig. 4c and d).

**Table 2:** Performance of the models in the identification of papillary thyroid carcinoma in Hashimoto’s thyroiditis patients

Cohort	Model	AUC (95%CI)	ACC	SEN	SPE	PPV	NPV
Training cohort	LR	0.762 (0.647–0.876)	0.718	0.796	0.593	0.761	0.640
	NB	0.792 (0.684–0.901)	0.761	0.909	0.519	0.755	0.778
	SVM	0.866 (0.776–0.956)	0.817	0.864	0.741	0.844	0.769
	MLP	0.796 (0.690–0.903)	0.718	0.705	0.741	0.816	0.606
Internal validation cohort	LR	0.778 (0.458–1.000.458.000)	0.778	0.750	0.833	0.900	0.625
	NB	0.819 (0.496–1.000.496.000)	0.833	0.833	0.833	0.909	0.714
	SVM	0.750 (0.428–1.000.428.000)	0.667	0.583	0.833	0.875	0.500
	MLP	0.778 (0.457–1.000.457.000)	0.722	0.667	0.833	0.889	0.556
External validation cohort	LR	0.736 (0.550–0.922)	0.756	0.750	0.769	0.875	0.588
	NB	0.690 (0.497–0.882)	0.659	0.607	0.769	0.850	0.476
	SVM	0.751 (0.589–0.914)	0.683	0.643	0.769	0.857	0.500
	MLP	0.783 (0.625–0.941)	0.732	0.643	0.923	0.947	0.546

*AUC Area under the receiver operating characteristic curve, *ACC *Accuracy, *SEN* Sensitivity, specificity, *CI* Confidence interval, *PPV* Positive predictive value, *NPV* Negative predictive value

**Fig. 5:**
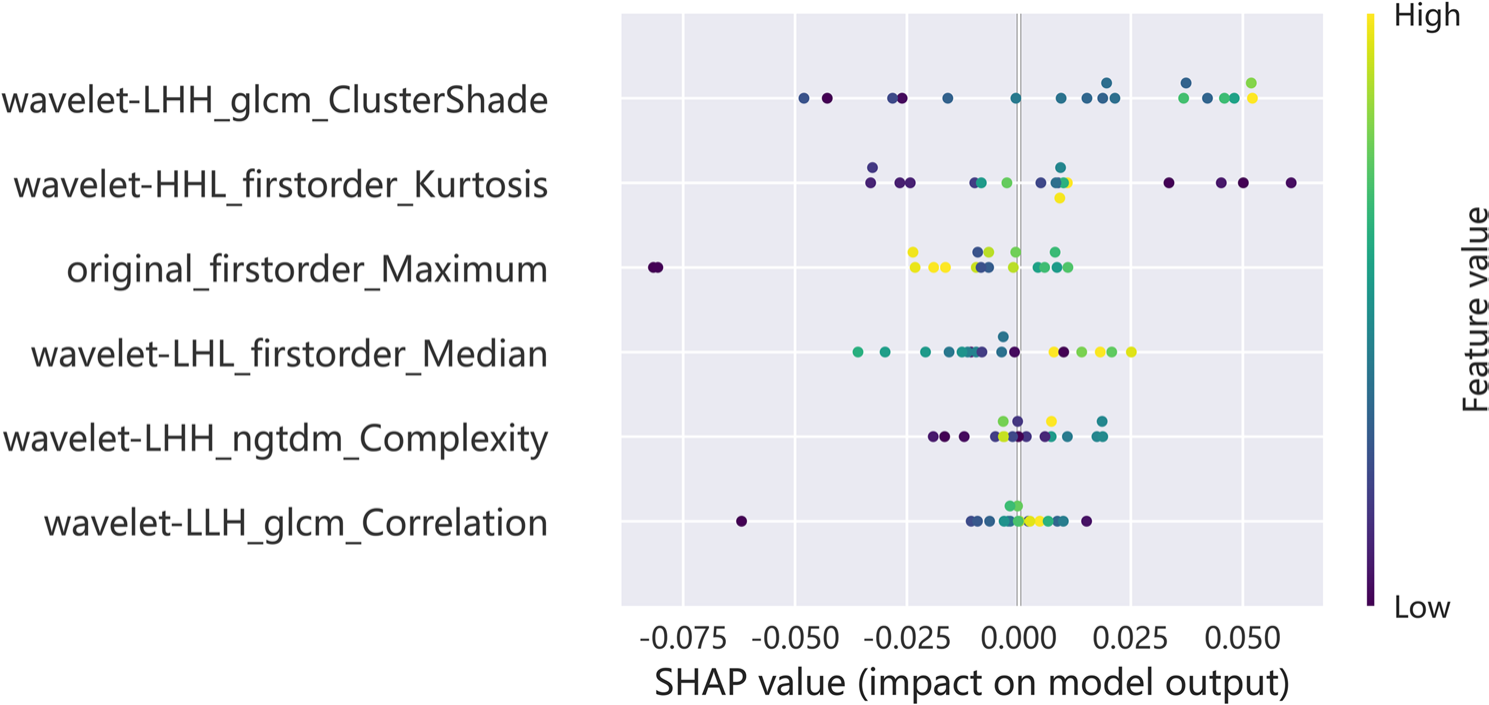
SHapley Additive exPlanations (SHAP) value distributions demonstrating feature importance rankings for radiomic features within the Multilayer Perceptron (MLP) predictive model

The SHAP map of the MLP is shown in Fig. 5. The SHAP analysis of MLP model highlighted several radiomic features that effectively predicted PTC in patients with HT, with wavelet-LHH_glcm_ClusterShade showing the highest importance, followed by wavelet-HHL_firstorder_Kurtosis, original_firstorder_Maximum, wavelet-LHL_firstorder_Median, wavelet-LHH_ngtdm_Complexity and wavelet-LLH_glcm_Correlation.

## Discussion

Identifying occult PTCs in HT is important for therapeutic decision making and for ensure timely, effective management. This study developed a radiomic model to predict the presence of PTC in patients with HT for early diagnosis. To our knowledge, this is the first study to predict the presence of PTC against the background of diffuse thyroid changes caused by HT using NECT radiomics. The MLP model had the best performance, with an AUC of 0.783 and a sensitivity and specificity of 0.643 and 0.923, respectively, in the external validation cohort.

Prior studies have supported the feasibility of radiomic approaches in PTC evaluation. Colakoglu et al. constructed a random forest model based on ultrasound texture features for distinguishing benign and malignant thyroid nodules with an AUC of 0.92 [14]. Some researchers have attempted to predict occult lymph node metastasis in clinically negative lymph node PTCs by establishing a model based on CT radiomic features, suggesting that the radiomic signature can predict lymph node status by reflecting the microstructure of the primary lesion [15]. Wang et al. reported that multiparametric MRI-based radiomics can accurately distinguish aggressive from nonaggressive PTC [16]. These studies provide evidence of the feasibility of radiomics in exploring the heterogeneity of thyroid lesions.

This study was not the first to analyze thyroid carcinoma in HT patients. Fang et al. proposed a radiomics model based on ultrasound to distinguish benign and malignant nodules in HT and achieved good results [12]; however, our study has several differences. First, we used NECT rather than ultrasound as the input. Both ultrasound and CT are commonly used imaging methods for evaluating the thyroid gland in clinical practice, but the quality of acquired ultrasound images is largely related to the differences in professional technical personnel’s personal experience, whereas CT is able to maximize the likelihood of acquiring consistent images through standardized scanning protocols [17]. Second, the previous study mainly extracted features from specific thyroid nodules. Considering that HT appears a diffuse lesion involving the entire thyroid gland, the present study extracted features of the entire thyroid and may be able to maximize the quantification of the gland in the context of HT.

Four ML models were constructed in this study. The MLP model had the best performance, with AUCs of 0.778 and 0.783 in the internal and external validation cohorts, respectively. The sensitivity, specificity, and accuracy of the MLP model in the external validation cohort were 0.643, 0.923, and 0.732, respectively. While the performance of this model did not fully meet our expectations, its high sensitivity suggests that it may help us find HT patients with suspected PTC. Furthermore, the increased use of chest CT, which is widely recommended for populations that require lung cancer screening, has resulted in increased detection of incidental thyroid disease [18–20]. The NECT radiomics models developed in this study also suggest potential utility for detecting PTC in patients with incidentally identified HT on CT performed for non-thyroid indications.

The SHAP analysis of MLP model highlighted several radiomic features that effectively predicted PTC in patients with HT. The observed features capture important aspects of tumor heterogeneity and structural alterations, where ClusterShade appears to reflect asymmetry in the gray-level co-occurrence matrix—potentially representing irregular spatial intensity distributions that may be associated with tissue disorganization, fibrosis, or microcalcification patterns. These imaging characteristics are thought to serve as established indicators of malignancy in HT and are likely accentuated during the process of malignant transformation. The prominence of wavelet-based features, particularly LHH and HHL decompositions, indicates that high-frequency texture variations are highly discriminatory, possibly reflecting microscopic infiltrative growth patterns or subtle calcifications that conventional imaging may miss [21]. Kurtosis measures the “peakedness” of voxel intensity distributions [22], highlighting focal high-intensity regions corresponding to areas of increased cellularity or microcalcification, while Maximum identifies the highest voxel intensities, potentially representing dense tissue or psammoma bodies characteristic of PTC [23]. Overall, the predominance of wavelet-derived features underscores the value of multi-scale textural analysis for detecting subtle malignancy-related alterations masked by the inflammatory background of HT.

This study has several limitations. First, because CT is not the first‑line modality for thyroid assessment, eligibility required a preoperative non‑contrast neck CT, which limited accrual despite a two‑center design (*n* = 130) and yielded a smaller cohort than many ultrasound‑based studies. Larger, prospective studies are needed to evaluate generalizability. Second, to ensure diagnostic certainty in identifying PTC coexisting within HT, we restricted inclusion to HT patients who underwent thyroid surgery and adopted postoperative histopathology as the reference standard rather than serologic markers, which are commonly used in clinical practice for diagnosing HT. While this strategy enhances internal validity for the target task, it likely introduced selection/verification bias by overrepresenting symptomatic patients or those with larger glands and, consequently, reduced the pool of eligible cases. Finally, our models were built solely from CT radiomics features; clinical variables (e.g., age, sex), laboratory data (including autoantibodies), and ultrasound findings were not incorporated and may further improve discrimination and calibration. Future work should integrate multimodal data and include external, prospective cohorts, particularly in settings where ultrasound remains the first‑line tool.

## Conclusion

Radiomics models based on non‑contrast neck CT may help identify PTC that may be obscured within diffuse HT, thereby supporting earlier diagnosis and intervention. Additionally, this NECT‑based approach may help flag PTC when HT is incidentally noted on neck CT and could extend to incidental thyroid findings on other CT examinations (e.g., chest CT), pending prospective validation.

## Data Availability

The data that support the findings of this study are available from the corresponding author on reasonable request.

## Abbreviations

HT: Hashimoto’s thyroiditis
PTC: Papillary thyroid carcinoma
CT: Computed tomography
ML: Machine learning
NECT: Nonenhanced CT
ICC: Intraclass correlation coefficient
LASSO: Least absolute shrinkage and selection operator
LR: Logistic regression
NB: Naive Bayes
SVM: Support vector machine
MLP: Multilayer perceptron
ANOVA: One-way analysis of variance
ROC: Receiver operating characteristic
AUC: Area under the curve
DCA: Decision curve analysis
SHAP: SHapley Additive exPlanations
